# Pseudolaric acid B attenuates atopic dermatitis-like skin lesions by inhibiting interleukin-17-induced inflammation

**DOI:** 10.1038/s41598-017-08485-x

**Published:** 2017-08-11

**Authors:** Zhen Yang, Meilun Liu, Wei Wang, Yiteng Wang, Bo Cao, Ying Gao, Hong Chen, Tan Li

**Affiliations:** 1 Department of Science Research, Logistics University of Chinese People’s Armed Police Force, Tianjin, 300309 P.R. China; 2 Department of Pharmacognosy and Pharmaceutics, Logistics University of Chinese People’s Armed Police Force, Tianjin, 300309 P.R. China; 3 Department of Urology, The Affiliated Hospital of Logistics University of Chinese People’s Armed Police Force, Tianjin, 300162 P.R. China; 4 Department of Pathogen Biology and Immunology, Logistics University of Chinese People’s Armed Police Force, Tianjin, 300309 P.R. China

## Abstract

Pseudolaric acid B (PB), isolated from the extract of the root bark of *Pseudolarix kaempferi Gordon*, has been used as a traditional remedy for the treatment of skin diseases. However, the information of PB on atopic dermatitis (AD) remains largely unknown. In the present study, oral administration with PB improved the severity scores of AD-like skin lesions dose-dependently in NC/Nga mice through reducing serum IgE, pro-inflammatory cytokines, and the infiltration of inflammatory cells. In addition, PB significantly attenuated the levels of IL-17 and IL-22, and the proportion of Th17 cells in NC/Nga mice, as well as decreased IL-17-induced inflammation in RAW264.7 cells. Moreover, PB inhibited the phosphorylation of IκBα and miR-155 expression both in NC/Nga mice and in IL-17-stimulated RAW264.7 cells, which could be reversed by GW9662, a specific antagonist for PPARγ. The incorporation of GW9662 reversed the inhibitory effect of PB on the RORγ-mediated activation of the *Il17* promoter. Transfection with PPARγ luciferase reporter gene further demonstrated the enhancement of PB on PPARγ transactivation. These findings indicate that PB could ameliorate AD-like skin lesions by inhibiting IL-17-induced inflammation in a PPARγ-dependent manner, which would provide experimental evidence of PB for the therapeutic potential on AD and other inflammatory skin diseases.

## Introduction

Atopic dermatitis (AD) is a kind of complicated, chronically relapsing inflammatory skin disease, characterized by edema, erythematous, scaly and lichenified lesions. The pathogenesis of AD has been attributed to a complex interaction including environmental factors, host susceptibility genes, altered skin barrier function, and immunologic abnormality (cutaneous hyper-sensitivity, immunoglobulin E (IgE)-mediated sensitization, and so on). This complexity has hindered the development of an efficacious AD treatment^1^.

Topical corticosteroids with strong anti-inflammatory properties achieve a faster improvement of AD, but their long-term use may produce a wide range of undesirable adverse effects, rebound phenomenon and intermittent recurrences^2^. Recently, several studies evaluating therapies based on natural substances as potential agents have suggested that patients with AD may be benefit from these raw materials^3^. One such agent, Pseudolaric acid B (PB), isolated from the extract of the root bark of *Pseudolarix kaempferi Gordon* (pinaceae), is a diterpene acid with a molecular structure that includes a compact tricyclic core containing a fused [5–7] ring system (polyhydroazulene), an unusual trans substitution pattern at the ring fusion site (C4–C10), and 4 contiguous stereocenters, including one quaternary (C10)^4^. These features suggest that PB may have broad pharmacological effects including anti-carcinogenesis, anti-angiogenesis, anti-microbial and anti-inflammatory activities^5, 6^. However, the information of PB on AD has not been reported until now, and the underlying molecular mechanism by which PB would antagonize inflammatory reaction remains largely unknown.

The NC/Nga mouse is the most commonly used disease model of AD showing clinical symptoms with erythema, scaling, itching and dryness spontaneous similar to those observed in AD patients, and has been the most extensively studied animal model of AD^7^. However, the low incidence of AD-like skin lesions, late onset of disease and poor reproducibility are its disadvantages^7^. To solve this problem, contact sensitizers such as 2,4-dinitrofluorobenzene (DNFB) would be adopted to induce AD-like skin lesions in NC/Nga mice. Repeated application of DNFB to the same skin site of NC/Nga mice could result in an immediate-type response followed by a late reaction, showing immunological alterations associated with the pathogenesis of AD^8^. Therefore, we decided to investigate the anti-inflammatory and immunoregulatory effects of PB using DNFB-induced murine model of AD in NC/Nga mice, and explored the underlying pharmacological mechanisms.

## Results

### PB ameliorates DNFB-induced AD-like clinical symptoms in NC/Nga mice

**Figure 1:**
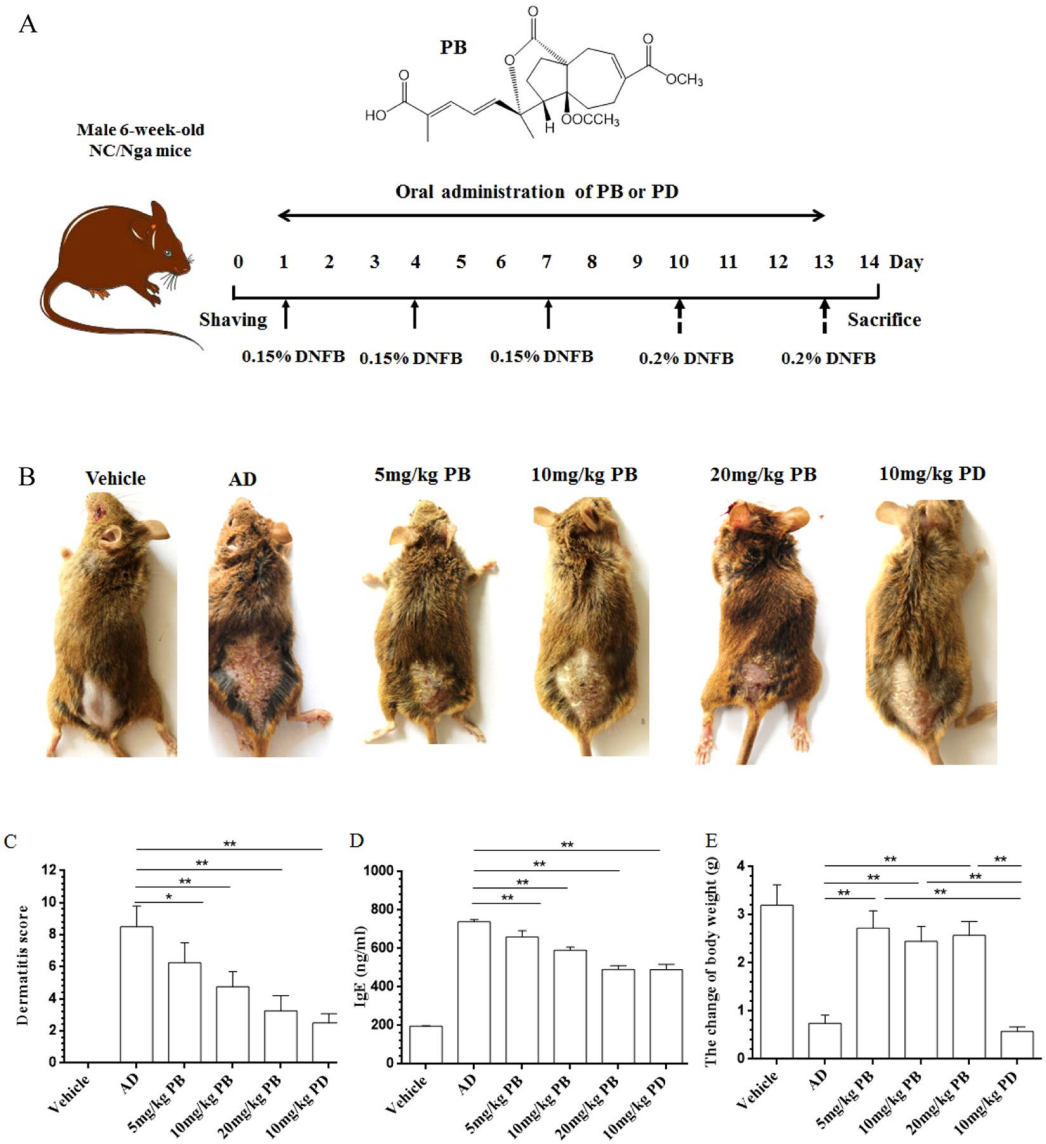
Improvement of PB on the clinical skin severity of AD-like skin lesions in NC/Nga mice. (**A**) Experimental protocol of AD-like lesions for sensitization and challenge with DNFB in NC/Nga mice. The NC/Nga mice were evoked by repetitive painting of 0.15% DNFB on dorsal skin once daily on days 1, 4 and 7, then further challenge with 0.2% DNFB on days 10 and 13. The treatment groups received PB (5, 10, 20 mg/kg) or PD (10 mg/kg) orally from days 1 to 13. (**B**) Representative dorsal skin photographs of each treatment group showing comparison of AD-like skin lesions. (**C**) Overall dermatitis score was determined from the sum of all individual scores. (**D**) The concentration of total IgE in serum. (**E**) The changes in body weight of mice. Data are representative of two independent experiments and presented as mean ± SD of n = 8 mice per group. *p < 0.05, **p < 0.01. Vehicle, intact mice with saline treatment; AD, DNFB-sensitized and challenged mice; PB, pseudolaric acid B; PD, prednisolone.

We firstly investigated the effect of PB on the relief of DNFB-induced AD-like symptoms in NC/Nga mice. As shown in Fig. 1, topical application of DNFB to the dorsal surface of NC/Nga mice could induce AD-like skin lesions and symptoms including erythema, erosion, scaling, edema, and lichenification, reaching a score of 11 points. However, oral administration with PB significantly relieved the severity scores of AD-like skin lesions in a dose-dependent manner. Elevation of serum IgE is one of the key characteristics of patients with AD, which may be used as a diagnostic and prognostic indicator for AD^9^. Thus, we also found that total serum IgE levels were significantly increased by repeated DNFB treatment in NC/Nga mice, which was attenuated by PB as well as prednisolone (PD), a well-known anti-inflammatory drug. At the end of the experiment, the change of body weight was measured to assess the general health status of mice. The results showed that oral application of PB markedly increased the body weight compared with AD group and PD group.

### PB inhibits inflammatory cells infiltration in NC/Nga mice

**Figure 2:**
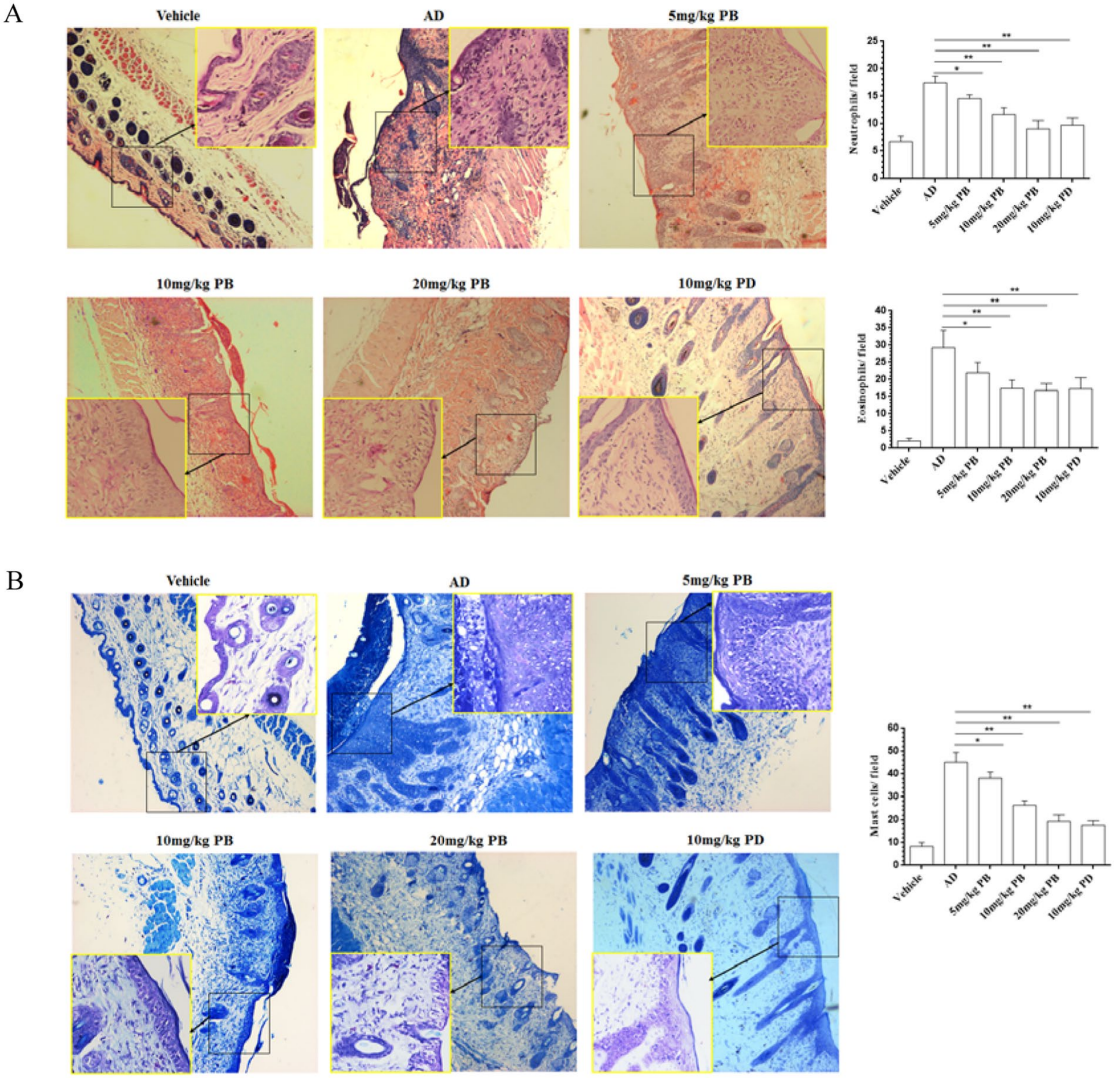
Histological features of skin tissues. (**A**) The sections from NC/Nga mice were stained with H&E staining. Representative dorsal skin photographs captured showing comparison of AD-like skin lesions. Magnification 40×, inset 200×. (**B**) The levels of mast cells by toluidine blue staining. Magnification40 ×, inset 200 ×. In these experiments, we used 3 slides per mouse, and observed 3 fields per slide. Data are representative of two independent experiments and presented as mean ±SD of n = 8 mice per group. *p < 0.05, **p < 0.01. Vehicle, intact mice with saline treatment; AD, DNFB-sensitized and challenged mice; PB, pseudolaric acid B; PD, prednisolone.

Marked histological changes including epidermal hyperplasia, hyperkeratosis, acanthosis and massive infiltration by inflammatory cells to just below the keratinocytes were observed in the skin lesions of NC/Nga mice, which could be markedly ameliorated by PB significantly (Fig. 2A).

Mast cells activated by allergen-sensitized IgE could induce the release of inflammatory cytokines and granular mediators correlated with the AD-like skin lesion development. In the present study, toluidine blue staining indicated the prominent number of mast cells in the dermal area of NC/Nga mice induced by DNFB, whereas PB considerably decreased these dermal changes. PD treatment also remarkably ameliorated the above alterations, similar to PB treatment (Fig. 2B).

### PB reduces pro-inflammatory cytokines levels

**Figure 3:**
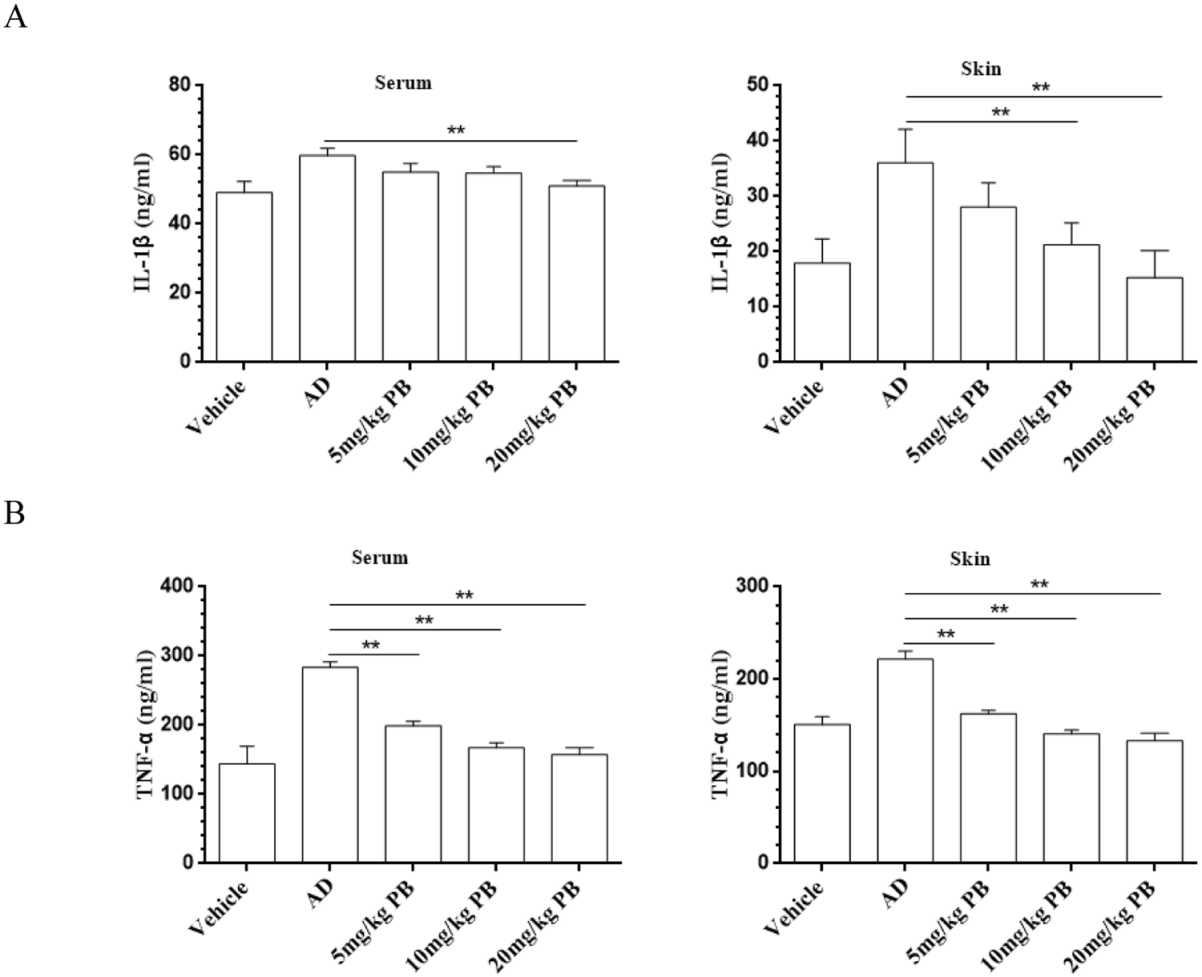
Suppression of PB on IL-1β and TNF-α in NC/Nga mice. The concentrations of IL-1β and TNF-α in serum and lesional skin tissues were detected by ELISA. (**A**) IL-1β. (**B**) TNF-α. Data are representative of two independent experiments and presented as mean ± SD of n = 8 mice per group. **P < 0.01. Vehicle, intact mice with saline treatment; AD, DNFB-sensitized and challenged mice; PB, pseudolaric acid B.

Then, we further examined the effects of PB on IL-1β and TNF-α, which are important to accelerate the development of AD^10^. The data showed that PB significantly decreased IL-1β and TNF-α release in the serum and lesional skin of AD mice (Fig. 3).

### PB suppresses IL-17 and IL-22 production

**Figure 4:**
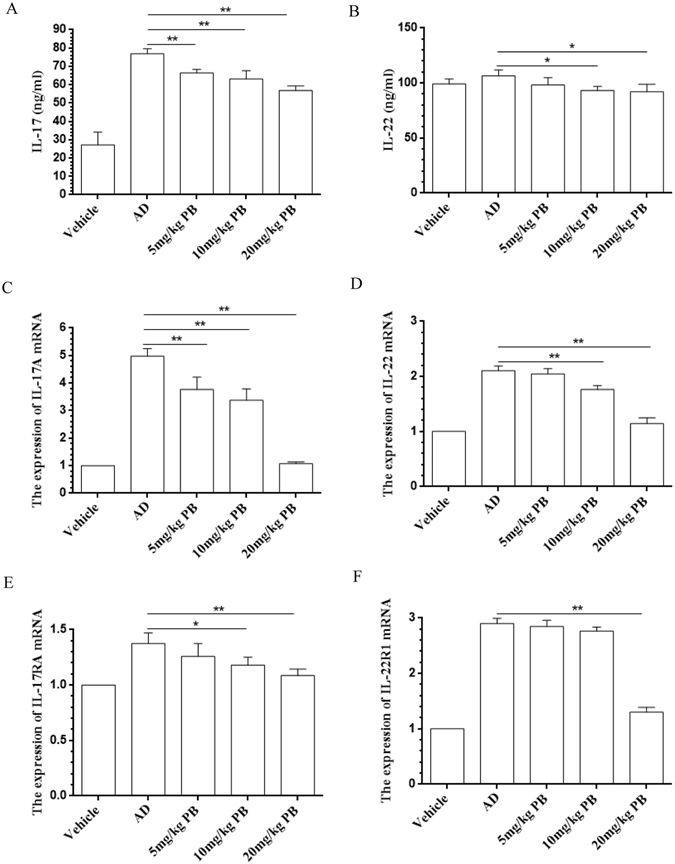
Inhibition of PB on IL-17 and IL-22 in NC/Nga mice. The concentration of serum IL-17 (**A**) and IL-22 (**B**) measured by ELISA. The expression of IL-17A (**C**), IL-22 (**D**), IL-17RA (**E**) and IL-22R1 (**F**) in the lesional skin tissues were evaluated by qRT-PCR and expressed as fold induction relative to vehicle. Data are representative of two independent experiments and presented as mean ± SD of n = 8 mice per group. *p < 0.05, **p < 0.01. Vehicle, intact mice with saline treatment; AD, DNFB-sensitized and challenged mice; PB, pseudolaric acid B.

Recently, Th17 and Th22 cells have been implicated in AD-related immune dysregulation by amplifying the inflammatory response^11^. IL-17 and IL-22 are main effective cytokines of Th17 and Th22 cells, which could induce the secretion of IL-1β, TNF-α and other pro-inflammatory cytokines^12, 13^. The functional IL-22 receptor consists of two receptor subunits, IL-22R1 and IL-10R2, among which IL-22R1 plays a much more important role than IL-10R2 during AD^13^. We thus evaluated the effects of PB on the levels of IL-17 and IL-22. As shown in Fig. 4, PB significantly decreased IL-17 and IL-22 in serum and down-regulated IL-17/IL-22-related genes expression including IL-17A, IL-17RA, IL-22 and IL-22R1 in the lesional skin of NC/Nga mice. However, the inhibitory effect of PB on IL-17 was much more significant than IL-22, prompting us to further explore the role of PB on IL-17.

### PB contributes to the decreased number of Th17 cells

**Figure 5:**
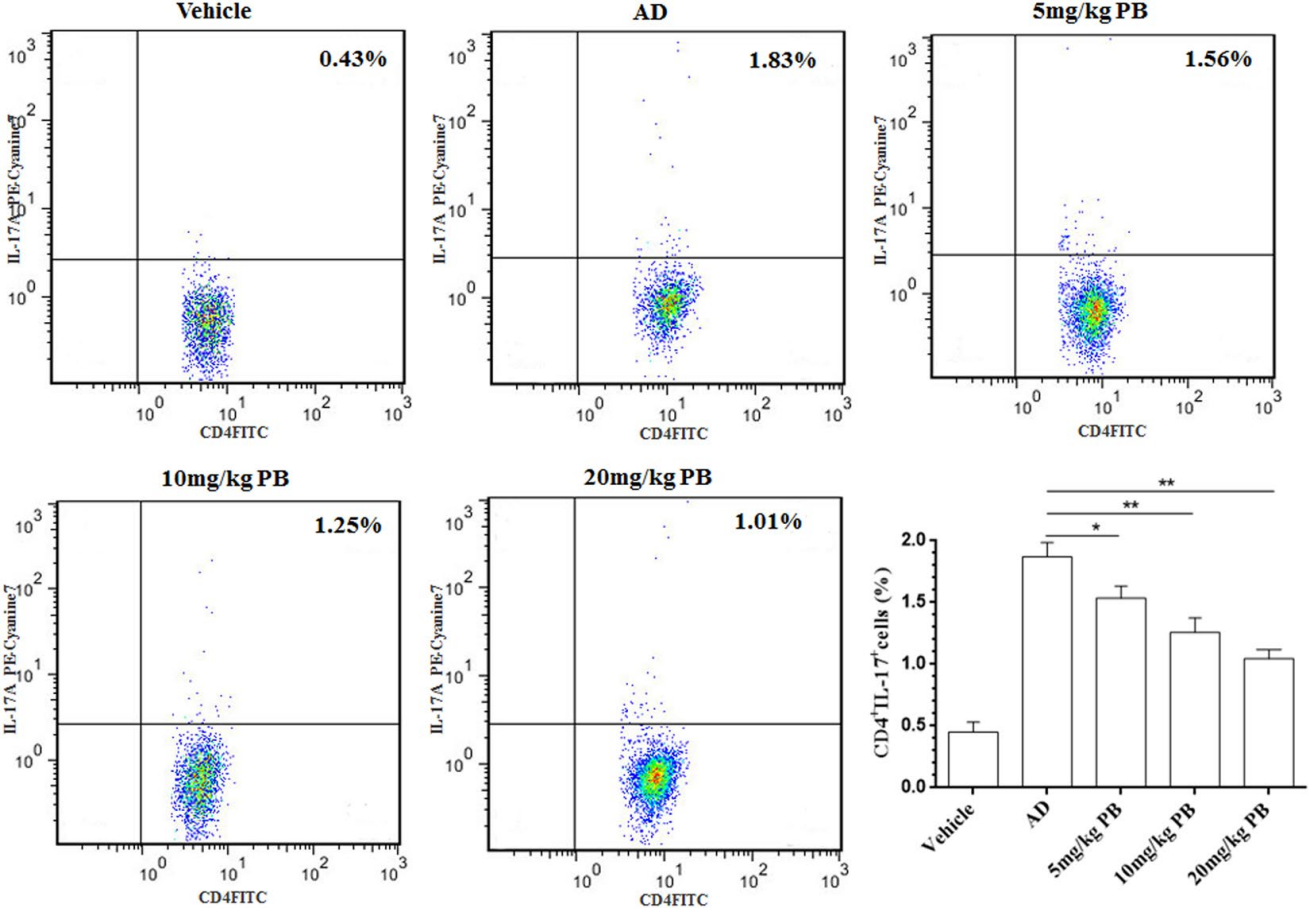
Down-regulation of PB on Th17 cells in NC/Nga mice. Cells collected from the draining lymph nodes of NC/Nga mice were stained and determined by flow cytometric analysis as showed in methods. Representative fluorescence-activated cell sorting plots showing the frequency of IL-17A-producing cells within the population of CD4^+^T cells. Numbers in the dot plots represented the percentages of Th17cells. Data are representative of two independent experiments and presented as mean ± SD of n = 8 mice per group. *p < 0.05, **p < 0.01. Vehicle, intact mice with saline treatment; AD, DNFB-sensitized and challenged mice; PB, pseudolaric acid B.

Several reports have demonstrated a marked increase in the IL-17^+^CD4^+^T-cell population (Th17) derived from AD patients compared with healthy controls, and the highest percentage of IL-17-producing CD4^+^T cells was found in severe AD, suggesting Th17 is the major cellular source of IL-17 in AD^14, 15^. Therefore, we examined the contribution of PB to the proportion of Th17 cells in the draining lymph nodes of NC/Nga mice. Consistent with previous findings on IL-17, vehicle group had significantly increased amounts of Th17 cells in AD mice, while treatment with PB decreased the percentage of Th17 cells dose-dependently (Fig. 5).

### PB attenuates IL-17-induced inflammatory response

**Figure 6:**
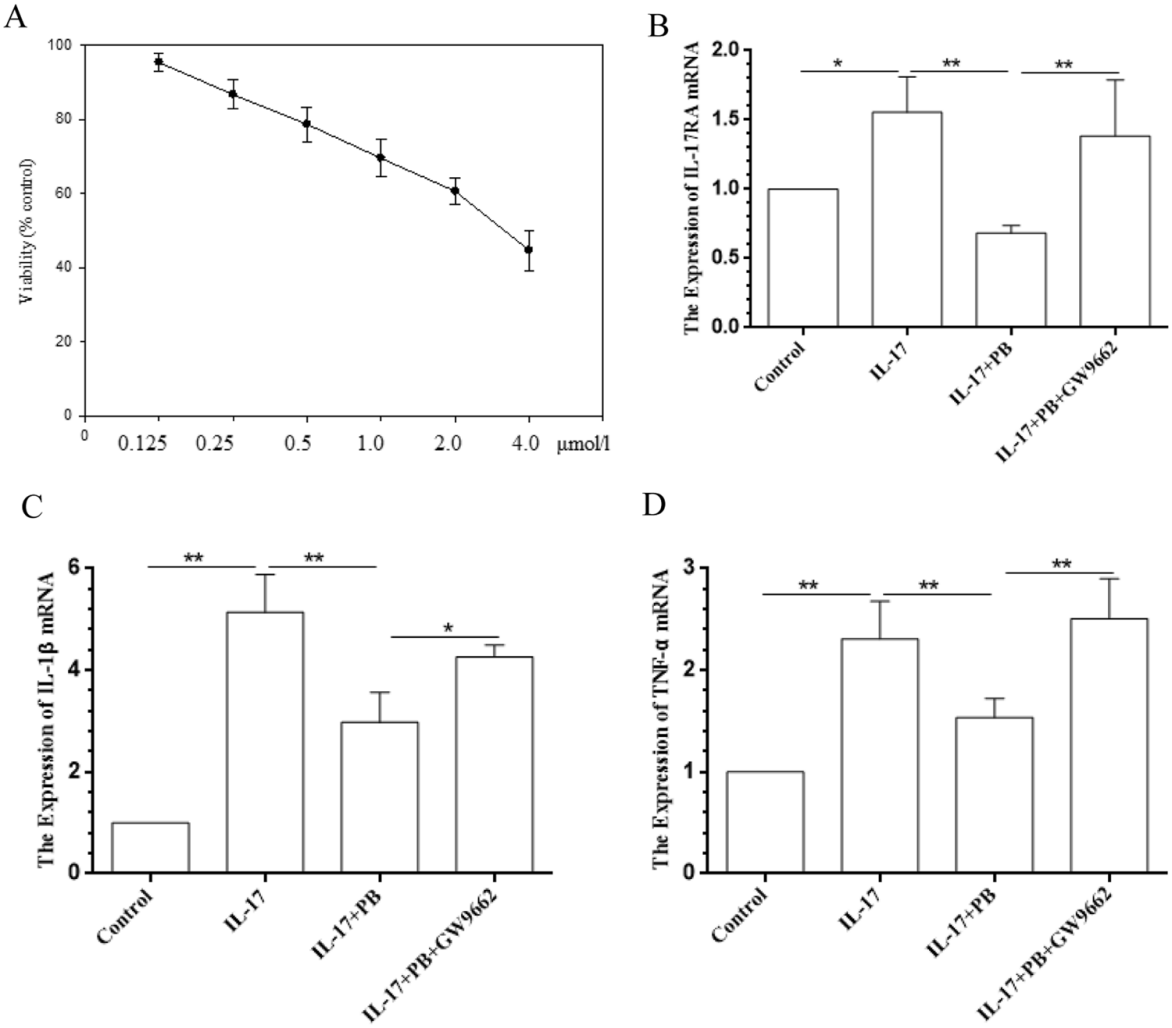
Effects of PB on IL-17-derived cytotoxicity and inflammatory response. (**A**) RAW264.7 cells were incubated with different concentrations (0, 0.125, 0.5, 1.0, 2.0, 4.0 μmol/l) of PB for 24 h and cell viability was determined by the CCK-8 assay. In another experiment, RAW264.7 cells were pre-incubated with GW9662 (1.0 μmol/l) for 1 h before added with PB (0.5 μmol/l). After 21 h, cultured cells were stimulated with IL-17 (10 ng/ml) for 3 h. The mRNA levels of IL-17RA (**B**), IL-1β (**C**) and TNF-α (**D**) were expressed as fold induction relative to control. Data are presented as mean ± SD. Each measurement was performed in duplicate and all experiments were repeated three times. *P < 0.05, **P < 0.01.

Macrophages are the source of many cytokines that play fundamental roles in pathogenesis of AD. Activated macrophages have a high capacity to produce inflammatory cytokines such as IL-1β and TNF-α^16^. Previously, we have proved that PB could reduce the mRNA expression of IL-1β and TNF-α in RAW 267.4 murine macrophage cells treated with LPS^17^. However, the action of PB on IL-17-induced inflammatory response remains to be demonstrated. Therefore, RAW264.7 cells were firstly incubated with increasing concentrations of PB for 24 h and subjected to the CCK-8 assay to assure whether PB would affect cell viability. As shown in Fig. 6A, PB decreased cell viability to 80% at a concentration of 0.5 μmol/l, higher concentrations of 1.0, 2.0 and 4.0 μmol/l decreased cell viability from 70% to 45%. Therefore, a non-cytotoxic concentration of PB (0.5 μmol/l) was chosen for further experiments. The results showed that PB at 0.5 μmol/l significantly reduced the expression of IL-17RA, IL-1β and TNF-α in RAW 264.7 cells stimulated with IL-17 (Fig. 6B~D).

### PB up-regulates Peroxisome proliferator-activated receptor γ (PPARγ) expression and activation

**Figure 7:**
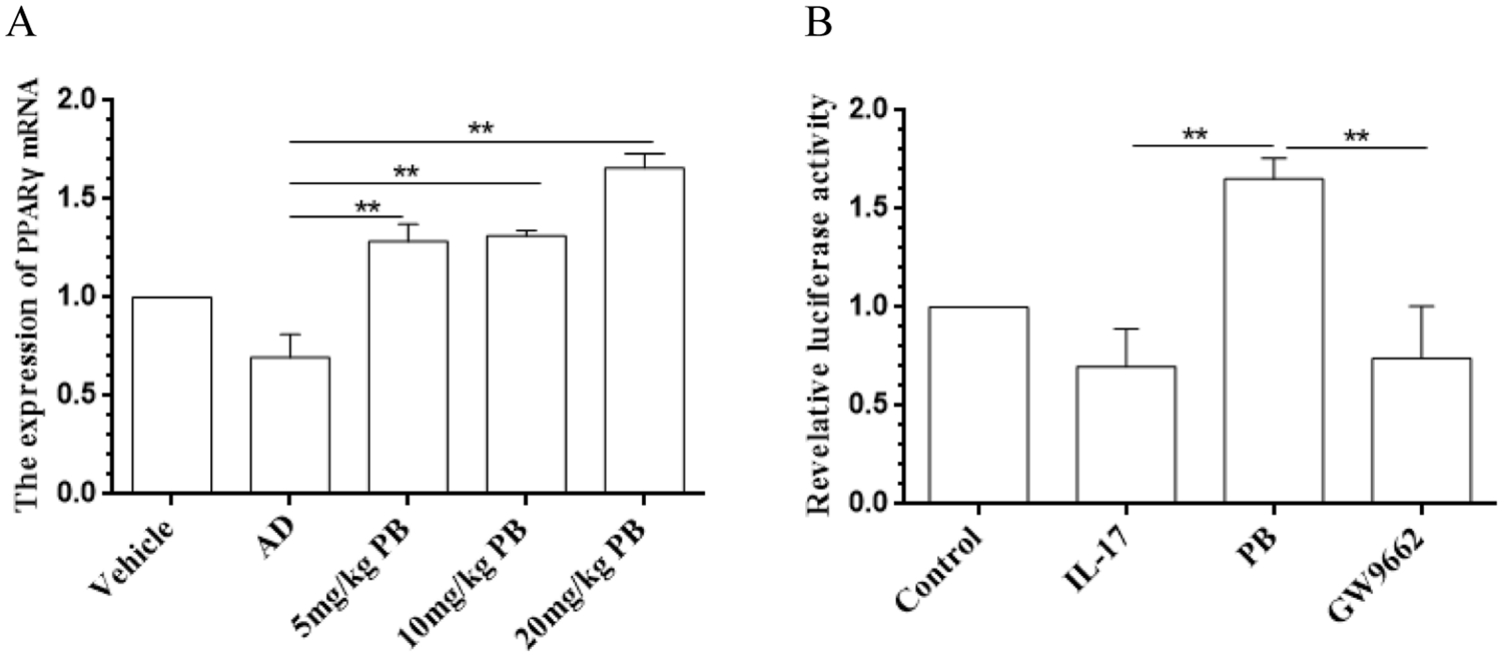
Enhancement of PB on PPARγ expression and activation. (**A**) The expression of PPARγ mRNA in the lesional skin tissues of NC/Nga mice was measured by qRT-PCR. Data are representative of two independent experiments and presented as mean ± SD of n = 8 mice per group. Vehicle, intact mice with saline treatment; AD, DNFB-sensitized and challenged mice; PB, pseudolaric acid B. (**B**) Induction of a luciferase reporter gene following ligand-dependent activation of a PPARγ fusion receptor in transiently transfected RAW264.7 cells as described in methods. The luciferase activity was measured and normalized by Renilla luciferase expression vector. Data are presented as mean ± SD. Each measurement was performed in duplicate and all experiments were repeated three times. **P < 0.01.

Considering that PPARγ is a negative regulator of IL-17 and could improve the clinical symptoms of AD^18^, we investigated the role of PPARγ using NC/Nga mice and IL-17-stimulated RAW264.7 cells to the best of our knowledge on PB. Figure 7A indicated that PB could indeed promote PPARγ expression in AD mice. Blocking PPARγ with GW9662, a specific antagonist for PPARγ, remarkably abolished the anti-inflammatory effects of PB in IL-17-induced RAW 264.7 cells (Fig. 6B~D). Moreover, PB could obviously enhance the transcriptional activity of PPARγ, which was also reversed by GW9662 completely (Fig. 7B).

### PB regulates RORγ-mediated *Il17* promoter activation

**Figure 8:**
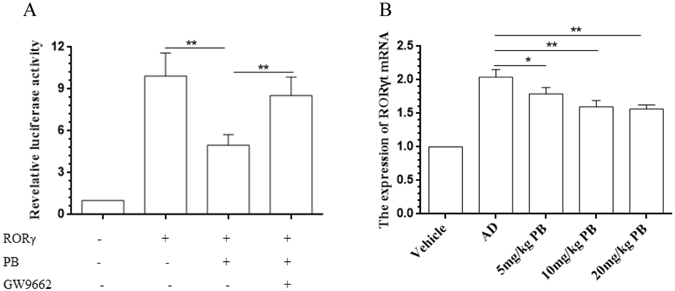
Inhibition of PB on RORγ-mediated *Il17* promoter activation. (**A**) Human T cell derived Jurkat cells were obtained from ATCC, and maintained in RPMI-1640 medium containing 10% FBS and antibiotics, which were co-transfected with pCMV-β-Gal, pCMV10-3xFlag-RORγ and pGL4.14 reporter plasmid under the control of *Il17* promoter and treated with PB at 0.5 μmol/l or pre-incubated with GW9662 (1.0 μmol/l) 1 h before added with PB. After 24 h, relative luciferase activity was determined and normalized against β-galactocidase activity. Data are presented as mean ± SD. Each measurement was performed in duplicate and all experiments were repeated three times. **P < 0.01. (**B**) Total RNA from the draining lymph nodes of NC/Nga mice was extracted and reverse transcribed into cDNA. The mRNA expression of RORγt was measured by qRT-PCR. Data are representative of two independent experiments and presented as mean ± SD of n = 8 mice per group. *p < 0.05, **p < 0.01. Vehicle, intact mice with saline treatment; AD, DNFB-sensitized and challenged mice; PB, pseudolaric acid B.

Retinoid-related orphan receptor gamma t (RORγt) has been reported to directly regulate the transcription of IL-17 and play a critical role in the differentiation of Th17^19^. Further, Th17 cells are controlled by their master transcriptional factor RORγt, which has been implicated as culprits during the immune response and inflammation of AD^20^. On the basis of the above data, PB could reduce IL-17 level and Th17 cells proportion remarkably. To further examine whether the effect of PB on IL-17 would be due to its regulation on transcriptional activation, we transfected Jurkat cells with RORγ expression plasmid under the control of the *Il17* promoter. The results showed that blocking PPARγ using GW9662 also reversed the inhibitory effect of PB on RORγ-mediated activation of *Il17* promoter, indicating that the inhibition of PB on *Il17* promoter activity might be PPARγ-dependent (Fig. 8A). Moreover, PB administration could also lead to a significant decrease for the expression of RORγt mRNA in the draining lymph nodes of NC/Nga mice (Fig. 8B).

### PB represses miR-155 expression

**Figure 9:**
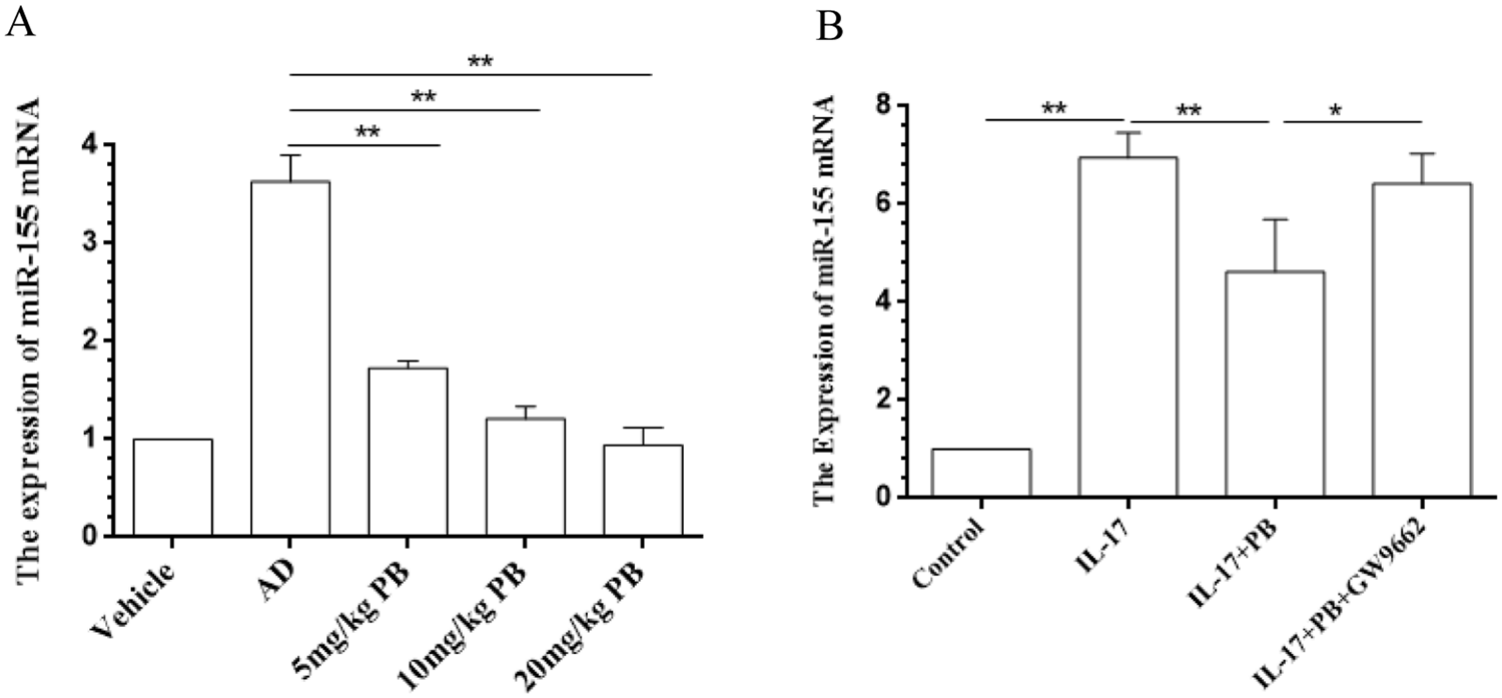
Regulation of PB on miR-155 expression. (**A**) Total RNA from the lesional skin tissues of NC/Nga mice was extracted and the expression of miR-155 was performed by qRT-PCR. The relative expression of miR-155 was normalized to the endogenous control U6. Data are representative of two independent experiments and presented as mean ± SD of n = 8 mice per group. Vehicle, intact mice with saline treatment; AD, DNFB-sensitized and challenged mice; PB, pseudolaric acid B. (**B**) RAW264.7 cells were pre-incubated with GW9662 (1.0 μmol/l) for 1 h before added with PB (0.5 μmol/l). After 21 h, cultured cells were stimulated with IL-17 (10 ng/ml) for 3 h. Cells were harvested and total RNA was used to quantify the level of miR-155 mRNA by qRT-PCR. Data are presented as mean ± SD. Each measurement was performed in duplicate and all experiments were repeated three times. *P < 0.05, **P < 0.01.

Based on the established anti-AD effects of PB and the important role of miR-155 during the development of AD through enhancing inflammatory response^21^, we thus proposed that PB might produce its beneficial action via affecting miR-155 expression. Figure 9A showed the expression of miR-155 was enhanced in the lesional skin from AD mice, which could be repressed by PB dose-dependently. In addition, miR-155 has a seed region at the 3′UTR of PPARγ gene, leading to regulate the PPRE signaling of PPARγ^22^. Our data confirmed that PB markedly decreased miR-155 expression in IL-17-sitmulated RAW 264.7 cells, as it was reversed by GW9662 (Fig. 9B).

### PB suppresses the NF-kB pathway

**Figure 10:**
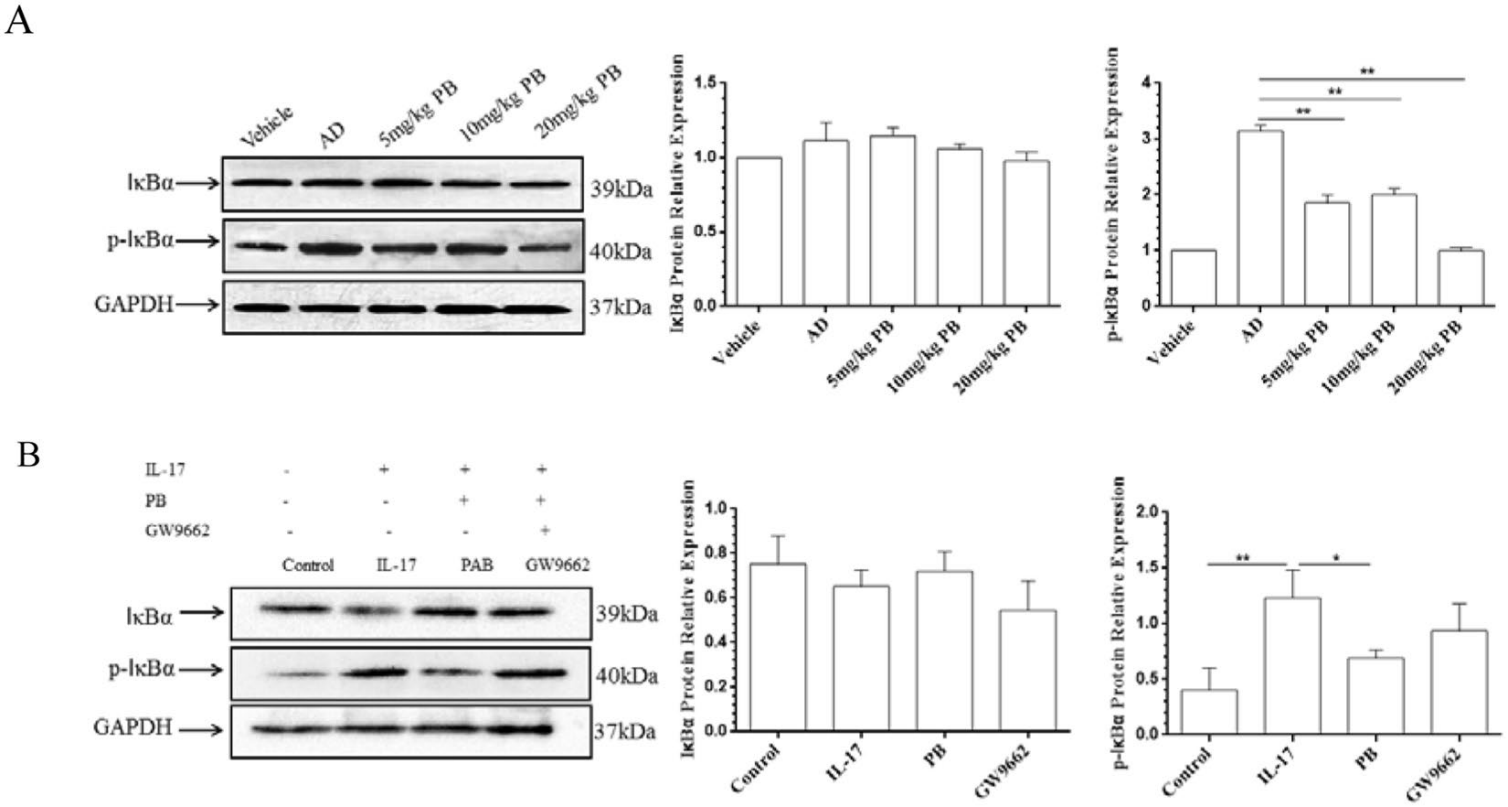
Effect of PB on the phosphorylation of IκBα. (**A**) The relative expression levels of IκBα and phospho-IκBα (p-IκBα) in the lesional skin tissues of NC/Nga mice were detected using western blot analysis. GAPDH was used as an internal control. Vehicle, intact mice with saline treatment; AD, DNFB-sensitized and challenged mice; PB, pseudolaric acid B. (**B**) RAW264.7 cells were pre-incubated with GW9662 (1.0 μmol/l) for 1 h before added with PB (0.5 μmol/l). After 21 h, cultured cells were stimulated with IL-17 (10 ng/ml) for 3 h. The phosphorylation of IκBα as well as its total protein levels in RAW264.7 cells were measured by western blot analysis. Densitometric analysis of IκBα and p-IκBα protein levels is represented as mean ± SD from 5 independent experiments. *P < 0.05, **P < 0.01.

NF-kB is a transcription factor that modulates the expression of many genes associated with inflammatory processes^23^. Li *et al*. reported that PB significantly inhibited the nuclear translocation of NF-kB p65 and the phosphorylation of IkBα in active T lymphocytes^24^. To further understand the roles of PB on NF-κB signaling during the development of AD, we measured the phosphorylation of IκBα and found that PB could markedly inhibit the activation of phospho-IκBα both in AD mice and in IL-17-stimulated RAW264.7 cells. The incorporation of GW9662 also reversed the inhibitory effect of PB on phospho-IκBα (Fig. 10).

## Discussion

AD is one of the most common chronic or chronically relapsing inflammatory skin diseases with a high prevalence, which could be considered to consist of acute and chronic phases. In the acute phase, infiltrated helper T cells (Th) and the levels of associated cytokines are increased, accompanied with IgE hyper-production by releasing histamine and several pro-inflammatory cytokines that cause pruritus in inflammatory skin lesions^25^. During the late-phase reactions, mast cells favor the recruitment of eosinophils and neutrophils. The complex and unexplored etiology of AD has made the development of therapeutics difficult. For the past few decades, anti-histamine agents, immunosuppressive agents, moisture care therapy, corticosteriods, or localized immunoregulatory agents have been used for treating AD^26^, and the whole body corticosteriods treatment is often chosen for severe cases of AD. However, long-term use of oral and topical corticosteriods is associated with a large number of side effects and drug intolerance^27^. Though new topical calcineurin inhibitors have been used for the treatment of AD recently, their efficacy was not sufficient to treat AD successfully^27^. Therefore, further evaluation is warranted to heighten the potential for developing safer and more efficacious therapies against AD. The application of herbal medicines has a long history due to their use in a folk medicine, and most well-known medications are derived from plants^28^, some of which have been reported as alternative therapeutics for anti-AD treatment^29^. Recent studies have reported that PB, isolated from herbal medicine pseudolarix, has a negative effect on T lymphocytes through inhibiting inflammation and pro-inflammatory factors, leading to a suppression of differentiation, proliferation, and inflammatory responses of T cells^6, 17, 30, 31^, which indicated PB might be regarded as an anti-inflammatory agent for the treatment of AD.

For objective assessment, we attempted to evaluate the therapeutic effects of PB using a multidisciplinary approach. Firstly, visual analysis was performed macroscopically using the clinical skin severity score for AD^32^. PB significantly resulted in the elimination of AD-like clinical symptoms in NC/Nga mice, including skin lesions and mast cell infiltration beneath hypodermis. Then, we focused on the effect of PB on IgE alteration, and found that PB treatment significantly inhibited serum IgE production. Moreover, PB markedly increased body weight compared to PD-treated mice and AD control, suggesting that oral application of PB was safe and effective.

It’s well known that inflammatory cell skin infiltrate is common in all AD lesions. The most frequent cell type make up the Th cells. A number of abnormalities play critical roles in the pathogenesis of AD including Th2-skewed cytokine expression in acute AD^33^. Afterwards, environmental triggers could provoke a mixed immune response of Th1 in the progression of AD and the resulting cytokine signaling elicited subsequent inflammation unrelated to the early Th2 response, suggesting Th1 cytokines such as TNF-α and IL-1β play a more dominant role than Th2 cytokines in the chronic status of AD^21^. Therefore, we focused on the effects of PB on TNF-α and IL-1β.The results showed that PB significantly reduced the levels of IL-1β and TNF-α in lesional skin tissues and serum of NC/Nga mice.

Th17 cells are characterized by the production of IL-17, which have been implicated in the pathogenesis of AD because IL-17 is crucial for the up-regulation of numerous inflammation-related genes in keratinocytes, fibroblasts, leading to the increased production of chemokines, antimicrobial peptides, and cytokines like TNF-α and IL-1β. These IL-17 activities could promote tissue fibrosis, chronicity of the inflammatory process, and the evolution of chronic inflammatory cutaneous lesions that contribute to the development of AD^11, 34^. Besides, Th22 cells represent a distinct T cell subset involved in epidermal immunity and remodeling, which also play very important roles during the pathogenesis of AD^35, 36^. The discovery of Th17 and Th22 cells has opened up a new avenue for research into the etiology and treatment of AD. In this study, PB obviously suppressed serum IL-17/IL-22 levels and the expression of IL-17/IL-22 related genes in the lesional skin tissues. However, PB might counteract inflammatory response much more through suppressing IL-17 than IL-22. Based on the above analysis, we thus investigated the mechanism of PB on IL-17. Our results showed that PB treatment significantly reduced IL-17-induced inflammatory response in RAW264.7 cells, indicating the therapeutic effects of PB on AD might be mediated by IL-17 partly. The ability of PB to inhibit IL-17 production and Th17 differentiation was further assessed in NC/Nga mice. These findings suggested that PB might act as an antagonist of IL-17 to attenuate AD-like skin lesions.

Peroxisome proliferators activated receptors (PPARs) belong to the nuclear receptor superfamily, which appear to be essential for maintaining skin barrier permeability, inhibit keratinocyte cell growth, promote keratinocyte terminal differentiation and regulate skin inflammation^18^. Four different subtypes have been identified: PPARα, PPARβ/δ and PPARγ. Recently, PPARγ and its agonists were found to have anti-inflammatory activity and immunoregulatory function in patients and animal models with both irritant and allergic skin diseases^37^. The activation of PPARγ could cause the repression of pro-inflammatory gene expression, and ameliorate AD-like skin lesion in NC/Nga murine model^38^. Moreover, PPARγ could selectively suppress Th17 differentiation via the inhibition of RORγt expression, confirming it as a negative regulator of RORγt^39^. Our previous studies have found that PB could promote PPARγ expression and affect the balance of Th17/Treg cells in a murine model of allergic contact dermatitis^6^. In the present study, we further demonstrated that PB could suppress RORγ-mediated *Il17* promoter activation in Jurkat cells, and inhibit RORγt expression in NC/Nga mice by enhancing PPARγ activation, which results suggested the amelioration of PB on AD-like skin lesions would be involved in its mechanism to suppress IL-17 through increasing PPARγ transactivation and activation.

MicroRNAs (miRNAs) are a novel class of short (21–25 nucleotides), non-coding, evolutionary conserved RNAs that regulate post-transcriptional gene expression through incomplete base pairing with the 3′-untranslated region (3′-UTR) of target mRNAs to degrade the target mRNAs or inhibit translation^40^. Numerous evidences have indicated that miRNAs are critically involved in regulating immune system, among which miR-155 plays an important role in inflammatory and immune reactions. Moreover, miR-155 has been reported to be over-expressed in the patients with AD through modulating the differentiation and function of Th17^21^. For further insight into the role of PB on the relationship between miR-155 and IL-17 during the pathogenesis of AD, we analyzed the expression of miR-155 in AD-like skin lesions of NC/Nga mice, and proved that PB exerted a significant inhibitory effect on miR-155 expression. Then, we further verified the inhibition of PB on miR-155 in IL-17-stimulated RAW264.7 cells. The results would provide a more detailed insight into the mechanism of PB in alleviating inflammation of AD.

NF-κB is a master switch of inflammatory gene expression and regulates the levels of IL-17, IL-1β, TNF-α and so on. Uncontrolled activation of NF-κB is associated with various human diseases including inflammatory skin diseases. In unstimulated cells, NF-κB is sequestered in the cytoplasm through interaction with inhibitory κB (IκB) proteins. In response to inflammatory stimuli, a cascade of phosphorylation events increases the activity of the IκB kinase (IKK) complex; IKK acts to phosphorylate IκBα, which results in its degradation by the proteasome and, consequently, its dissociation from the NF-κB dimers. The release of IκBα promotes the translocation of NF-κB subunits to the nucleus, where it initiates transcription of pro-inflammatory genes^41^. Recent studied reported that miR-155 and IL-17 could aggravate the pathological process of AD by activating NF-κB signaling pathway^21, 42^. We and several pieces of literatures have correlated the anti-inflammatory effects of PB with the down-regulation of NF-κB pathway^6, 24, 30, 43^. In the present study, PB was confirmed to ameliorate AD-like skin lesions through a PPARγ-dependent NF-κB pathway.

**Figure 11:**
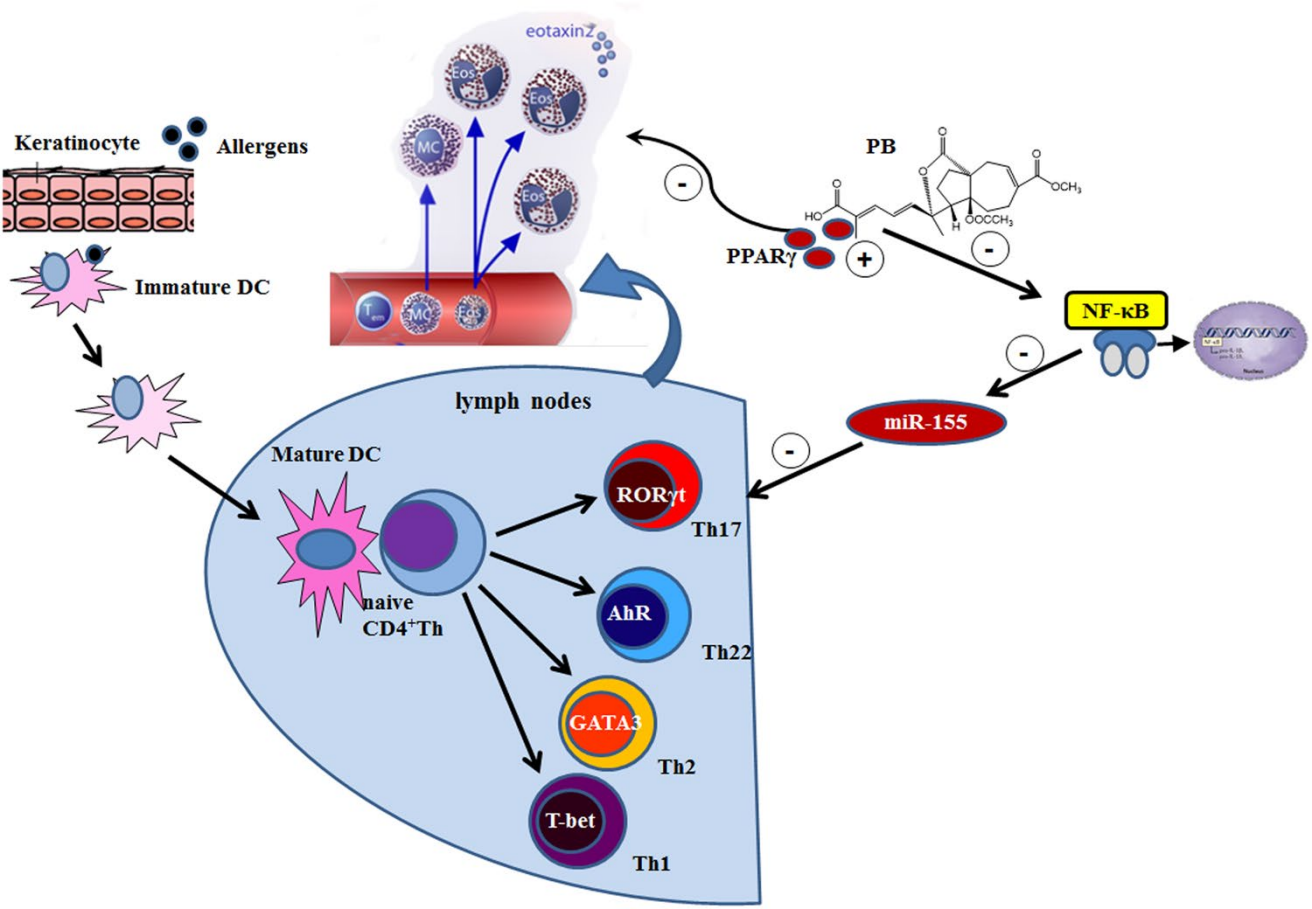
Scheme summarizing the potential mechanisms by which PB affects AD.

In conclusion, we have demonstrated the therapeutic efficacy of PB in an AD-like murine model, which mechanisms might be related to inhibit IL-17-induced inflammation, block NF-κB pathway and diminish miR-155 expression in a PPARγ-dependent manner (Fig. 11). Thus, the successful treatment of AD skin lesions is likely to require the use of drugs like PB with broad targets rather than the specific inhibition of single molecule. However, future studies are needed to define more precisely the mechanism of PB responsible for AD. Our results raise the possibility that PB could be a potential therapeutic candidate of herbal origin for AD and other inflammatory skin diseases.

## Materials and Methods

### Materials

PB was provided by professor Chen with extraction by 95% ethanol, column chromatography by EPE (ether: petroleum ether = 1:5) and recrystallization in methanol. The purity of PB was >98% determined by HPLC analysis^30^. Dulbecco’s modified Eagle’s medium (DMEM), RPMI-1640 medium, and fetal bovine serum (FBS) were purchased from Gibco BRL (Grand Island, NY, USA). PD, DMSO, DNFB (≥99% pure), monensin, ionomycin, phorbol-12-myristate-13-acetate (PMA) and GW9662 were purchased from Sigma-Aldrich (St. Louis, MO, USA). Anti-mouse CD4-FITC and IL-17A-PE-Cyanine7 antibodies were purchased from eBioscience (San Diego, CA). Anti-mouse IκBα, and phospho-IκBα (Ser32) antibodies were purchased from Cell Signaling Technology (Danvers, MA, USA). CD3ε mAb, GAPDH and horseradish peroxidase (HRP)-conjugated secondary antibodies were purchased from Abcam Technology. Mouse interleukin (IL)-17, IL-22, IL-1β and tumor necrosis factor α (TNF-α) Enzyme-linked immunosorbent assay (ELISA) kits were purchased from R&D Systems Inc. (Minneapolis, Minn., USA), and IgE ELISA kit was from Shibayagi Co. (Shibukawa, Japan). pRL-TK, pGL4.14 reporter plasmid, and Dual Luciferase II reporter assay kit were purchased from Promega (Promega Corp., Madison, WI, USA). Enhanced chemiluminescence (ECL) kit and bicinchoninic acid (BCA) protein assay kit were purchased from Pierce Biotechnology.

### Animals and experimental design

Specific pathogen-free male 6 weeks old NC/Nga mice were obtained from Riken BioResource center, Japan. Mice were maintained on a dark/light cycle (12/12 h) in air-conditioned rooms (22.5 ± 0.5 °C, 50 ± 5% humidity) and were adapted to local conditions for one week before the beginning of the study. All animal experiments were conducted according to national and international laws and policies (Guide for the Care and Use of Laboratory Animals) and were approved by the Institutional Animal Care and Use Committee of Logistics University of the Chinese People’s Armed Police Force.

As shown in Fig. 1A, DNFB sensitization were evoked by the topical application of 100 μl of 0.15% DNFB dissolved in acetone/olive oil (3:1, v/v) to the shaved dorsal skin of NC/Nga mice once on days 1, 4 and 7. On days 10 and 13, sensitized mice were challenged with 100 μl of 0.2% DNFB to the dorsal skin surfaces. A total of 48 NC/Nga mice were randomly divided into six groups with 8 mice in each: vehicle group (intact mice with saline treatment containing 0.5% DMSO), AD model group (DNFB-sensitized and challenged mice), three PB group (5 mg/kg, 10 mg/kg, 20 mg/kg), and PD positive group (10 mg/kg). PB was dissolved in DMSO as a stock solution and then diluted with saline media (DMSO ≤ 0.5%) to the indicated concentrations prior to use.

### Evaluation of dermatitis severity

The severity of AD-like symptoms was evaluated by the SCORAD method^44^, and features such as development of edema, erythema, oozing, crust, excoriation, and lichenification were scored as 0 (none), 1 (mild), 2 (moderate) or 3 (severe). Overall dermatitis score (minimum 0, maximum 12) was determined from the sum of all individual scores. Assessment was performed by an investigator who was blinded to the treatment groups.

### Histopathological analysis

By the end of the study period, mice were sacrificed and their lesional skin tissues were harvested and fixed with 4% paraformaldehyde in phosphate buffered saline (PBS, pH 7.4). Skin sections (3–5 μm) were stained with hematoxylin and eosin (H&E) for histopathological analysis and with toluidine blue for analysis of mast cells^45^. All images were captured with an Olympus BX41 microscope equipped with a video camera and analyzed using Image–Pro-Plus software (version 6.0, Media Cybernetics, MD, USA). A morphological evaluation of all the skin sections was carried out in a blinded fashion. Numbers of inflammatory cells and mast cells were expressed as average total counts in four fields of 100 μm^2^.

### ELISA

The serum of mice was collected and stored at −80 °C until use. Moreover, the lesional skin tissues of mice were separated and homogenized with ice-cold lysis buffer containing protein inhibitor. The protein concentration of the supernatant was measured using the BCA protein assay kit according to the manufacturer’s instructions. The levels of IgE, IL-17, IL-22, IL-1β and TNF-α were measured using ELISA kits according to the manufacturer’s protocol. The absorbance was measured at 450 nm using a microplate reader. A concentration below the lowest detectable value of the ELISA standard curve was assigned the mean value of the lowest concentration of the ELISA standard curve and zero.

### Quantitative real-time polymerase chain reaction (qRT-PCR)

Total RNA from lesional skin tissues was isolated using the TRIzol reagent (Invitrogen, Carlsbad, CA, USA) according to the manufacturer’s instruction. The purity of RNA samples was was assessed by inspecting the 28 S and 18 S bands after 1.5% agarose gel electrophoresis; a 260/280 absorbance ratio was between 1.9 and 2.0. Total RNA was transcribed into cDNA using a reverse transcription system (Promega, Madison, WI, USA), and the primers were purchased from Shanghai Sangon Biological Engineering Technology Company (Table 1). Amplification conditions were set to heat activation at 95 °C for 10 min, and followed by 40 cycles of denaturation at 95 °C for 15 s and annealing at 60 °C for 1 min. All melting curve analysis was performed between 50 °C and 95 °C. The qRT-PCR reactions were performed using the PerfeCTa SYBR Green SuperMix (Quanta BioSciences) on an ABI PRISM 7300 sequence detection system (Applied Biosystems, USA). The expression of target genes was normalized to β-actin, and the fold change of each sample was calculated using the comparative CT method (2^−△Ct^). All reactions were run in triplicates, and all experiments were performed three times.

**Table 1:** Primer sequences in this study.

Gene name	Primer sequence
β-actin	Forward: 5′-CTAAGGCCAACCGTGAAAAG-3′
	Reverse: 5′-ACCAGAGGCATACAGGGACA-3′
IL-17A	Forward: 5′-ACTACCTCAACCGTTCCACG -3′
	Reverse: 5′-TTCCCTCCGCATTGACACAG-3′
IL-17RA	Forward: 5′-TGGGATCTGTCATCGTGCT-3′
	Reverse: 5′-ATCACCATGTTTCTCTTGATCG-3′
IL-22	Forward: 5′-CAACTTCCAGCAGCCATACA-3′
	Reverse: 5′-GTTGAGCACCTGCTTCATCA-3′
IL-22R1	Forward: 5′-TGACCTTTCAACCCCTACGC-3′
	Reverse: 5′-TGAGGTCAGACAGGCTCTGC-3′
miR-155 (RT)	5′-GTCGTATCCAGTGCAGGGTCCGAGGTATTCGCACTGGATACGACCCCTAT-3′
miR-155	Forward: 5′-GCGCGTTAATGCTAATTGTGAT-3′
	Reverse: 5′-GTGCAGGGTCCGAGGT-3′
PPARγ	Forward: 5′-GAAAGACAACGGACAAATCACC-3′
	Reverse: 5′-GGGGGTGATATGTTTGAACTTG-3′
RORγt	Forward: 5′-GTCTGCAAGTCCTTCCGAGAG-3′
	Reverse: 5′-ATCTCCCACATTGACTTCTG-3′
U6	Forward: 5′-GCGCGTCGTGAAGCGTTC-3′
	Reverse: 5′-GTGCAGGGTCCGAGGT-3′

For microRNA-155 (miR-155) detection, RNA was isolated using the QIAGEN miRNeasy mini kit according to the manufacturer’s instructions (QIAGEN Inc., Valencia, CA, USA). Reverse transcription reaction was performed with specific miRNA primers (Table 1). All reactions were performed using TaqMan® MicroRNA Reverse Transcription Kit (Applied Biosystems, USA) following the manufacturer’s protocol. U6 snRNA was used as a reference gene to normalize the miR-155 expression.

### Fluorescence-activated cell sorting (FACS) analysis

Cells from the draining lymph nodes of mice were prepared as described previously^6^. Red blood cells were lysed with erythrocyte lysis buffer (0.15 M NH_4_Cl, 10 mM NaHCO_3_ and 0.1 mM EDTA; pH 7.3). The cell phenotype of Th17 cells was defined using the appropriate antibodies according to the manufacturer’s protocol. Briefly, the cells (2 × 10^5^ cells) were stimulated with immobilized anti-CD3ε mAb (2 μg/ml) for 6 h and incubated with 10 μg/ml Brefeldin A, 50 ng/ml PMA, and 1 mmol/l ionomycin. The stimulated cells were then stained with FITC-conjugated anti-CD4 antibody, fixed with 4% paraformaldehyde, and made permeable with permeabilization wash buffer. Finally, the cells were stained with PE-Cyanine7-conjugated anti-IL-17A mAb. Isotype controls were used to enable correct compensation and to confirm antibody specificity. The data were acquired on a FACScan flow cytometer (CytomicsTM FC 500, Beckman Coulter, USA) and analyzed using the FlowJo7 software (Treestar, Ashland, OR, USA).

### Cell culture and cytotoxicity analysis

RAW 267.4 cells were obtained from the American Type Culture Collection (ATCC, TIB-71), and maintained in DMEM supplemented with 10% FBS, 100 U/ml penicillin, and 100 mg/ml streptomycin and then plated into 12-well plates. PB was dissolved in DMSO and the final DMSO concentration was <0.1% in all experiments *in vitro*. To examine whether PB could affect cell viability, RAW264.7 cells were incubated with increasing concentrations of PB (0, 0.125, 0.25, 0,5, 1.0, 2.0, 4.0 μmol/l) for 24 h. Cytotoxic effect of PB was estimated by using Cell Counting Kit-8 (CCK-8; Dojindo Laboratories, Kumamoto, Japan) following the manufacturer’s instructions.

### Measurement of pro-inflammatory cytokines expression

For pro-inflammatory cytokine assay, RAW264.7 cells were incubated with PB (0.5 μmol/l) for 21 h and stimulated with 10 ng/ml of recombinant murine IL-17 (PeproTech Inc., Rocky Hill, NJ) for 3 h. Then, total RNA was extracted from RAW264.7 cells to determine the expression of IL-1β and TNF-α mRNA. For experiments involving PPARγ, RAW267.4 cells were pre-incubated with GW9662 (1.0 μmol/l) for 1 h before the addition of PB.

### Transient transfection and reporter gene assay

PPARγ transactivation assay was done to explore the mechanism of PB based on the previously protocol with some modifications^30^. PPARγ-induced transcriptional activity was evaluated by transient transfection of a TK-PPRE3x luciferase reporter plasmid driven by the PPRE-containing acyl-CoA oxidase promoter (kindly provided by Dr. R.M. Evans, The Salk Institute, Howard Hughes Medical Institute, CA). RAW264.7 cells were seeded in 6-well culture plates (2 × 10^6^cells/well). 24 h after seeding, cells were transiently transfected with 0.5 μg of TK-PPRE3x and 0.05 μg of pRL-TK using X-tremeGene™ HP (Roche, Mannheim, Germany) and OPTI-MEN (Invitrogen, Carlsbad, CA, USA) according to the manufacturer’s instruction. Six h later, the transfection medium was replaced with DMEM medium containing no PB, or PB (0.5μmol/l), or GW9662 (1.0 μmol/l) for 1 h before PB incubation. Cells were then cultured for 21 h, and were stimulated with IL-17 (10 ng/ml) for 3 h. Finally, the cells were lysed and the luciferase activities were determined with a luminometer according the instruction of Dual Luciferase II reporter assay kit. The values of luciferase activity were normalised with the pRL-TK values to correct the differences caused by unequal transfection efficiency. All values were normalized to control wells to calculate relative luciferase activity.

### Activation assay of the *Il1*7 promoter

To examine the effect of PB on the activation of the *Il17* promoter, Jurkat cells were co-transfected with pCMV-β-Gal plasmid (Clontech, Mountain View, CA), pCMV10-3xFlag-RORγ plasmid, and a pGL4.14 reporter plasmid (Promega Corp., Madison, WI, USA) under the control of human *Il17*-3kb-CNS promoter^46^, and then treated with vehicle, or PB (0.5μmol/l), or GW9662 (1.0 μmol/l) for 1 h before PB incubation. After 24 h, cell extracts were lysed and the firefly luciferase and β-galactocidase activities were measured using a Luciferase Assay Substrate kit (Promega) and a Luminescent β-galactosidase Detection kit II (Clontech), respectively.

### Western blotting analysis

The lesional skin tissues of mice and RAW264.7 cells treatment with IL-17 were collected and lysed in RIPA lysis buffer lysis buffer (50 mM Tris-HCl, pH 7.5, 150 mM NaCl, 1% NP-40, 1 mM DTT) supplemented with a protease inhibitor mixture on ice. 50 *μ*g lysates were quantified using a BCA protein assay kit and separated on a 12% SDS-polyacrylamide gel electrophoresis (PAGE) for western blotting as described previously. Transferred blots were then incubated overnight at 4 °C with the anti-phospho-IκBα (Ser32) (1:500), followed by HRP-conjugated goat anti-rabbit secondary antibody for 2 h (1:3000). The transferred proteins were visualized with an ECL detection kit using autoradiography films. After removing the labeling signals by stripping buffer (62.5 mM Tris-HCl, pH 6.7, 100 mM 2-mercaptoethanol, 2% SDS) at 50 °C for 30 min, the membrane was reprobed with anti-IκBα antibody by the same experimental procedures. Specific bands were scanned and quantified using the Quantity One software (Bio-Rad). GAPDH was used as the internal control.

### Statistical analysis

Data were presented as mean ± standard deviation (SD) and were analyzed using one way analysis of variance (ANOVA) followed by Turkey-Kramer test or two tailed Student t-test when appropriate. All analyses were performed using GraphPad Prism version 6.0 Software (San Diego, CA, USA). A value of *p* < 0.05 was considered statistically significant.
